# Perioperative Fluoroquinolone Treatment Deteriorates Prognosis Following Coronary Artery Bypass Grafting

**DOI:** 10.3390/jcdd9060173

**Published:** 2022-05-28

**Authors:** Min Zhang, Lijuan Jian, Xinping Min, Bowen Li, Xin Cai, Zhiwei Wang, Zhipeng Hu

**Affiliations:** Department of Cardiovascular Surgery, Renmin Hospital of Wuhan University, 238 Jiefang Road, Wuhan 430060, China; zhangmwhurm@whu.edu.cn (M.Z.); jianlijuan@whu.edu.cn (L.J.); minxinping@sina.com (X.M.); bowenli@whu.edu.cn (B.L.); rm000640@whu.edu.cn (X.C.); wangzhiwei@whu.edu.cn (Z.W.)

**Keywords:** coronary artery disease, coronary artery bypass grafting, fluoroquinolone, side effect, survival

## Abstract

Background: Former studies have revealed that fluoroquinolone (FQ) can induce aortic expansion and rupture. While FQ is widely used in perioperative anti-infection therapy, its impact on graft patency and patient survival is unknown. Methods: Coronary artery bypass grafting (CABG) data were extracted from the MIMIC-III database. Chi-square tests, Fisher’s exact tests, *t*-tests, or ANOVAs were used to compare baseline data between groups determined by FQ therapy status, depending on the data type. Propensity score matching was used to establish a balanced cohort. Cox regression was used to investigate the impact of FQ on CABG patient survival, whereas paired *t*-tests were used to analyze secondary results. Results: Of the 5030 patients who underwent CABG, 937 (18.6%) received oral or intravenous FQ therapy. Using propensity score matching, these 819 patients were successfully matched with 819 controls in a 1:1 ratio. Cox regression showed that FQ significantly decreased survival among CABG patients (HR: 1.62, 95% CI: 1.21–2.15, *p* = 0.001). Furthermore, FQ usage was associated with longer hospitalization (<0.0001), ICU duration (<0.0001), ventilation period (<0.0001), and duration of vasopressor administration (<0.0001). Conclusions: Perioperative FQ therapy was associated with worse prognosis and a more difficult recovery among patients with CABG.

## 1. Introduction

Coronary heart disease (CHD) is common among the elderly and is increasingly prevalent in the middle-aged population [1]. Coronary artery bypass grafting (CABG) is the most effective treatment for severe CHD; however, the operation results in significant operative trauma [2]. Unavoidable complications include graft occlusion (GO) and graft thrombosis (GT), both of which significantly decrease perioperative and long-term survival [3].

Fluoroquinolone (FQ) administration was recently identified as a risk factor for several connective tissue diseases, such as acute aortic dissection, aortic aneurysm, retinal detachment, and tendinopathies [3,4,5,6,7]. These diseases share several important pathogenesis features with GO, such as the remodeling of the extracellular matrix, inflammation, and the abnormal expression of metalloproteinases. The aorta and tendons are strong connective tissues, but could be susceptible to deterioration due to the effects of FQ with catastrophic consequences [8]. Patients administered FQ within 60 days have an increased risk of suffering aortic dissection, indicating an acute FQ effect on blood vessels [9]. Coronary arteries and grafts are connective tissues forming part of the vascular system. However, whether FQ affects GT and GO was not investigated.

Therefore, we conducted a propensity score matching study of CABG patients using the MIMIC-III (Medical Information Mart for Intensive Care) database [10,11], and found that FQ decreased the prognosis of CABG patients. We also found that FQ usage prolonged the hospital stay of patients, the period in the ICU, the requirement for vasopressors, and ventilation duration.

## 2. Methods

### 2.1. Ethics Declaration

This study utilized de-identified data from the MIMIC-III database with pre-existing institutional Review Board (IRB) approval. The project was approved by the institutional Review Boards of the Massachusetts Institute of Technology (MIT) and the Beth Israel Deaconess Medical Center (BIDMC) and was granted a waiver of informed consent. Thus, approval from our institution (Renmin Hospital of Wuhan University) was exempted. Patients or the public WERE NOT involved in the design, or conduct, or reporting, or dissemination plans of our research.

### 2.2. Data Extraction

The data of patients receiving CABG in Beth Israel Deaconess Medical Center, from 1 June 2001 to 31 October 2012, were extracted from the MIMIC-III database by Pgadmin4 using icd-9 procedure codes (openly available for download to registered users). Complications were also extracted by icd-9 codes, whereas age was calculated by the admission date minus the birth date. Hospitalization duration was calculated by subtracting the admission date from the discharge date, and ICU stay duration was calculated by subtracting the date of entering the ICU from the day the patient left. Vasopressor duration was calculated from the time that one or more vasopressors (including norepinephrine, epinephrine, vasopressin, dobutamine, and milrinone) were administered to the patient. Patients before 2008 were followed up for at least nine months, while those between 2008 and 2012 were followed up for at least four years. Survival time was calculated by subtracting the date of admission from the date of death. According to the third international consensus definitions for sepsis and septic shock, sepsis was defined as a condition resulting in life-threatening organ dysfunction caused by a dysregulated host response to infection [12]. 

### 2.3. Statistical Analysis

Patients were categorized into groups (FQ and non-FQ) based on whether they received FQ treatment. As the local application of FQ in the eyes or ears involves a very low dosage, the three patients who accepted this treatment method were assigned to the non-FQ group. 

When comparing baseline data, categorical variables were expressed as counts and percentages and were compared between the FQ and non-FQ groups using Chi-square or Fisher’s exact tests. Continuous variables are expressed as mean (standard deviation) or median (interquartile range (IQR)) and compared using the *t*-test between the two groups, or by using one-way ANOVA among three or more groups when the data conformed to a normal distribution. Otherwise, non-parametric tests were performed [13]. 

Propensity score matching was performed according to the method and R function outlined by Daniel et al. (method = “nearest”, ratio = 1, caliper = 0.2) [14]. Briefly, the propensity score was estimated by running a logit model. The European System for Cardiac Operative Risk Evaluation (EuroSCORE) was the primary factor, and the covariates included other factors, such as smoking and hyperlipidemia. The propensity score was then calculated, and a histogram was generated. A matching algorithm, based on the target treatment (FQ) and covariates, was used to find pairs of observations with similar propensity scores. A love plot was generated using the “cobalt” package, whereas the paired *t*-tests or chi-square tests were used to test the balance in the matched cohorts. The covariates used for propensity matching included sex, age, admission type (ELECTIVE, EMERGENCY, URGENT), smoking, COPD, hyperlipidemia, diabetes, infectious endocarditis, neurological dysfunction, extracardiac arteriopathy, angina, history of cardiac surgery history, history of coronary intervention, pulmonary hypertension, plus other heart surgery (including valve surgery (replacement or repair)), congenital heart disease correction, pericardial adhesiolysis), plus aorta surgery, myocardial infarction, ejection fraction (30−, 30–50, 50+), dialysis status, and sepsis. A Cox model was used to investigate the impact of FQ on the survival of patients who received CABG. When performing Cox regression, we chose covariates based on consultation with expert cardiovascular surgeons for their clinical perspectives, and a comparison with the EuroSCORE model. Secondary results, such as duration of hospital or ICU stays, the requirement of vasopressors, and ventilation duration, were compared using paired *t*-test followed by covariance tests. Post-operative stroke, the IABP application was compared with Chi-test and/or logistic regression.

All statistical analyses were performed using R (Version3.6.3) and Rstudio (Version 1.2.5033, RStudio, Inc., Boston, MA, USA) software. Microsoft Excel 2015 was used to store and convert some of the data. This study was reported following the STROBE guidelines.

## 3. Results

### 3.1. Baseline Data of Study Cohort

A total of 5030 patients were included in this study, of which 937 (18.6%) accepted FQ therapy. Table 1 shows the baseline characteristics of the patients in the FQ and non-FQ groups. Not surprisingly, the percentage of patients who suffered sepsis, those having other synchronous heart or aortic surgery, and patients who experienced complications such as infectious endocarditis was much higher in the FQ group. Factors influencing FQ prescription included male gender and advanced age, and comorbidities, such as neurological dysfunction, extracardiac arterial diseases, pulmonary arterial hypertension, acute myocardial infarction, hyperlipidemia, and heart failure.

### 3.2. Primary Outcomes in the Matched Cohorts

**Figure 1:**
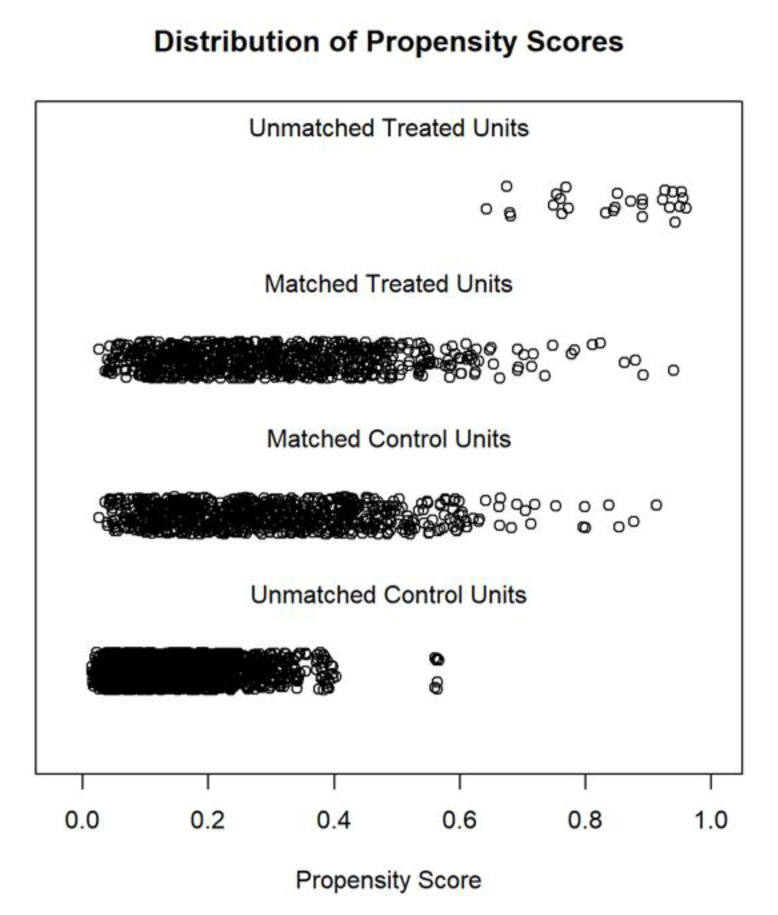
Propensity score distribution of the primary and the matched cohort.

**Figure 2:**
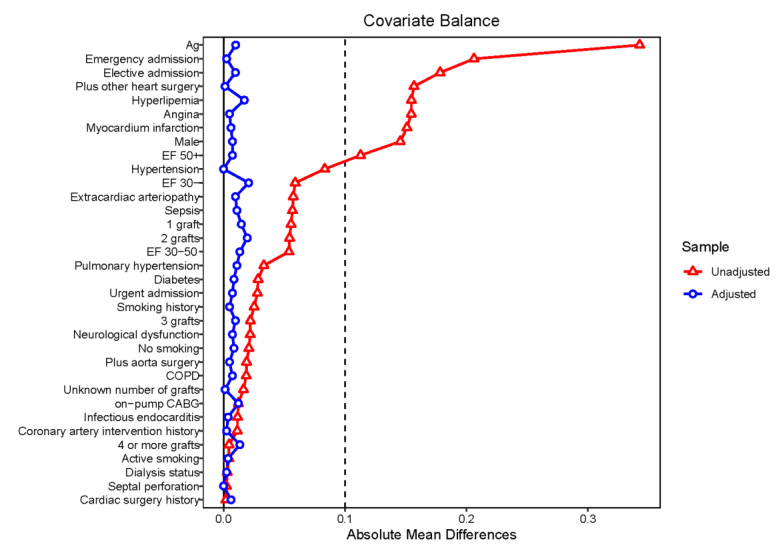
Balance of the matched cohort (love-plot generated by “cobalt” R package).

Propensity score matching was performed with a “nearest” strategy in a 1:1 ratio. Eight hundred and nineteen patients in the FQ group were matched with 821 patients in the non-FQ group. The differences in the baseline data between the groups were significantly reduced (Table 2). The propensity score distribution is shown in Figure 1 and the matched cohort balance is shown in Figure 2.

**Figure 3:**
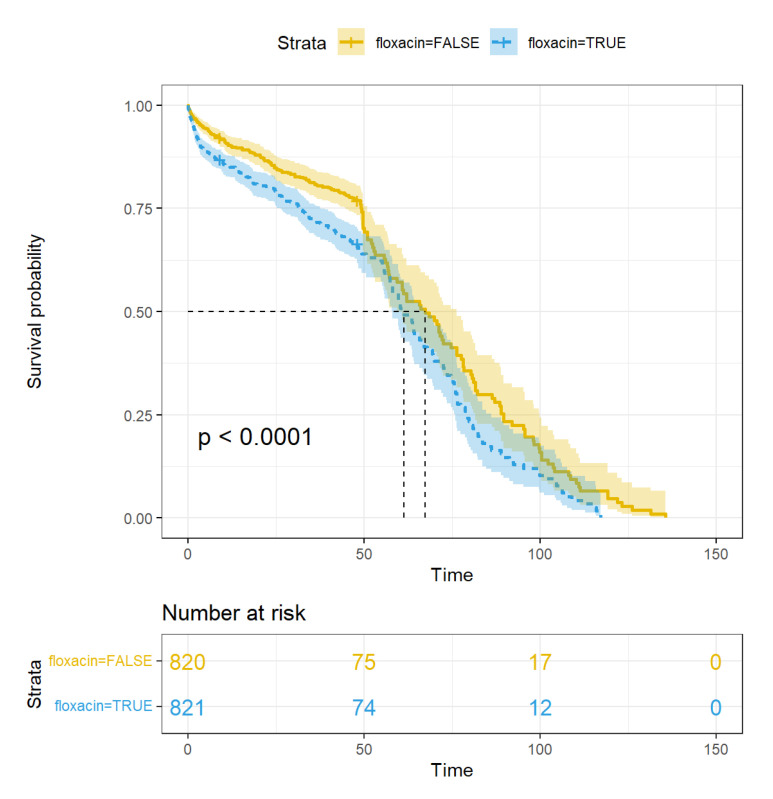
Kaplan–Meier survival curve of patients in FQ and non-FQ group: patients in FQ group haves worse survival than non-FQ group.

Cox regression was used to estimate the impact of FQ and other factors on patient survival. Treatment with FQ significantly decreased survival among the CABG patients (HR: 1.51, 95% CI: 1.26–1.81, *p* = 0.0000; Table 3; Figure 3). Other factors influencing survival included COPD (HR: 1.39, 95% CI: 1.11–1.75, *p* = 0.0047), coronary intervention history (HR: 0.68, 95% CI: 0.47–0.98, *p* = 0.0406), normal ejection fraction (HR:0.68, 95% CI 0.58–0.96, *p* = 0.0228), hyperlipemia (HR: 0.77, 95% CI: 0.63–0.93, *p* = 0.0068), sepsis (HR: 1.68, 95% CI: 1.15–2.47, *p* = 0.0077), and age (HR: 1.03, 95% CI: 1.02–1.04, *p* = 0.0000). The median follow-up was equal (48 months) in the two groups of the matched cohort.

### 3.3. Secondary Outcomes in the Matched Cohorts

Several key differences in secondary outcomes were observed (Table 4). In the matched cohort, 1591 patients survived until the time of discharge, 1619 patients survived until out of the ICU, and 1634 patients survived until disconnected from the ventilators. Specifically, FQ treatment was associated with longer hospitalization (*p* < 0.0001), periods in the ICU (*p* < 0.0001), ventilation duration (*p* < 0.0001), and vasopressor requirements (*p* < 0.0001) (Table 4). FQ treatment is associated with a higher incidence of suffering from in hospital death (*p* = 0.005), post-operative stroke (*p* = 0.012), and application of IABP (*p* = 0.0011) (Table 5).

**Figure 4:**
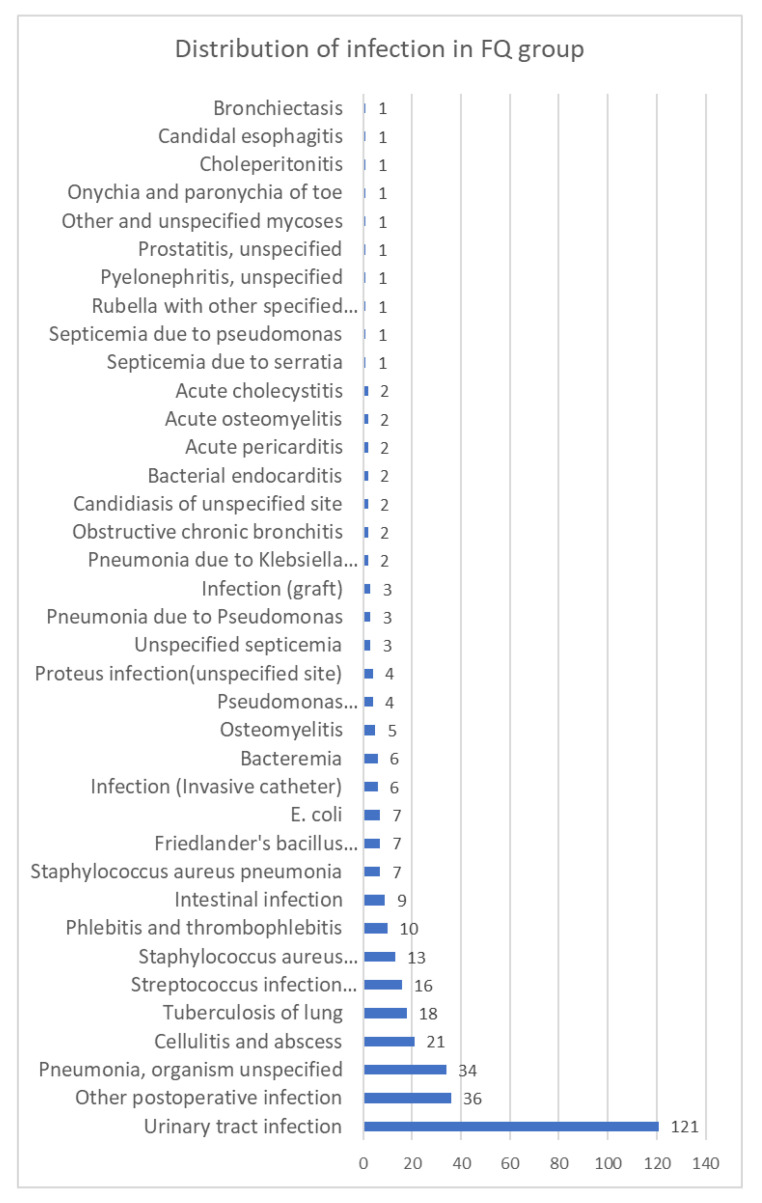
Distribution of infection in FQ group (some patients were diagnosed for several infections).

The distribution of infections is given in Figure 4. The most common infections include urinary tract infections, pneumonia, cellulitis, and abscess.

## 4. Discussion

Both preoperative conditions and perioperative therapies are determining factors of the clinical outcomes for CABG patients [15]. Therefore, the EuroSCORE system was established for estimating the impact of preoperative complications, whereas the syntax scoring system is used for estimating the impact and severity of coronary artery stenosis [16,17]. Anti-infection treatments are an important and common therapy during the post-operative period, and CABG patients are no exception. The effectiveness and safety of antibiotics are strictly tested in preclinical studies. However, preclinical studies often overlook special populations, such as CABG patients. To date, the impact of antibiotics on vascular diseases was not studied in depth. Concerningly, the impact of perioperative FQ treatment on the prognosis of CABG patients remains unknown.

Patients receiving FQ treatment in this study were more likely to suffer from sepsis, required synchronous heart or aortic surgery, and were complicated with infectious endocarditis. As FQ is an antibiotic prescribed only for bacterial infections that are more likely after complex and more invasive procedures, it is important to rule out the possibility that the increased mortality among the patients receiving FQ treatment is as a result of infection. Therefore, sepsis was set as a covariate in the present analysis. Both univariate and multivariate Cox regression confirmed that FQ was associated with reduced survival. From the survival curve, we found that the patients in the FQ group not only had worse survival in the hospital, but also in the longer term. This finding indicated that, even after patients receiving FQ therapy were discharged from the hospital, they had poorer survival. Meanwhile, we found that sepsis was not an independent risk factor for the poorer survival of CABG patients. As such, this study provides strong evidence that decreased survival rates in patients receiving FQ treatment are as a result of using FQ, and not as a result of infection.

Propensity score matching is useful in descriptive studies because it can balance multiple factors in existing data. This is especially important when the effect of a specific factor is to be investigated [18,19,20]. Many factors influencing CABG outcomes were identified before, whereas the impact of FQ was never studied. The data from the MIMIC-III database was unbalanced in many aspects. Propensity score matching achieved a balanced dataset to specifically focus on the side effects of FQ treatment. As a result, this has also confirmed that neurological dysfunction, cardiac surgery history, pulmonary artery hypertension, synchronous heart or aortic surgery, myocardial infarction, ventricular septal perforation, and sepsis are associated with reduced survival among CABG patients. These findings are in accordance with those from previous research.

It is unclear why FQ could induce vessel injury, and basic research regarding this aspect is currently lacking. FQ plays an antibacterial role by inhibiting DNA gyrase, thus impeding the normal replication, transcription, transportation, and recombination of DNA. It is possible that FQ was toxic to DNA and could affect essential biological processes, such as extracellular matrix expression and degradation, metalloproteinase expression, fibrotic activity, and collagen expression. 

EuroSCORE is a tool for predicting CABG outcomes [16]. The covariables of the present analysis were selected based on the EuroSCORE. Almost all factors in the EuroSCORE could be extracted from the MIMIC-III database, except for preoperative emergency conditions. Admission type was used, instead of preoperative emergency conditions. We extracted the left ventricular ejection fraction (LVEF) data from echo reports and discharge summaries using a regular formula-based natural processing strategy. In total, 4312 LVEFs were extracted. The percentage of missing data was 14.3%, which may account for why some patients in the FQ group were not successfully matched. Some important factors, such as smoking and hyperlipidemia, were also included in the analysis because they affected CABG outcomes. Based on its strong impact on the aorta, we hypothesized that FQ may increase coronary artery graft occlusion. However, we were unable to obtain direct evidence about post-operative graft patency from this database. Graft patency is the most important factor contributing to patient survival. We hold the opinion that the most plausible reason for the reduction in CABG survival is accelerated graft occlusion.

To further assess the impact of FQ on CABG patients, we also investigated the duration of hospitalization, the period in the ICU, vasopressor requirements, and ventilation duration. These results are sensitive in estimating the difficulty of the recovery process [18]. The results are consistent with our previous analysis and further confirm that FQ treatment perioperatively impairs CABG patient outcomes.

Our research highlights the risk of FQ treatment and reminds heart surgeons to avoid using FQ following CABG where possible, as well as following coronary interventions, peripheral and visceral vascular interventions, and cerebrovascular surgery and interventions. Treatment with FQ is not rare in CABG patients, yet there are many alternative antibiotic options that could be used. Prospective studies and basic experimental research should be conducted to further assess the risks of FQ in coronary artery surgery, as well as in other vascular diseases.

## Figures and Tables

**Table 1:** Baseline data of extracted patients from MIMICIII database. COPD: chronic obstructive pulmonary disease; LVEF: left ventricular ejection fraction.

Covariates	Non-FQ	FQ	*p*	SMD
*n*	4093	937		
Male (%)	3143(76.8)	592 (63.2)	<0.001	0.3
Age (mean (SD))	67.25(10.74)	70.99(10.78)	<0.001	0.348
Admission type (%)			<0.001	0.438
ELECTIVE	1603 (39.2)	214 (22.8)		
EMERGENCY	2268 (55.4)	706 (75.3)		
URGENT	222 (5.4)	17 (1.8)		
Smoking (%)			0.033	0.098
Active smoking	291 (7.1)	72 (7.7)		
No smoking	3363 (82.2)	791 (84.4)		
Smoking history	439 (10.7)	74 (7.9)		
Hypertension (%)	2781 (67.9)	555 (59.2)	<0.001	0.182
COPD (%)	563 (13.8)	150 (16.0)	0.083	0.063
Hyperlipemia (%)	2678 (65.4)	459 (49.0)	<0.001	0.337
Diabetes (%)	1545 (37.7)	379 (40.4)	0.134	0.055
Infectious endocarditis (%)	6 (0.1)	11 (1.2)	<0.001	0.127
Neurological dysfunction (%)	69 (1.7)	36 (3.8)	<0.001	0.132
Extracardiac arteriopathy (%)	419 (10.2)	145 (15.5)	<0.001	0.157
Angina (%)	1800 (44.0)	263 (28.1)	<0.001	0.336
Cardiac surgery history (%)	30 (0.7)	5 (0.5)	0.664	0.025
Coronary intervention history (%)	426 (10.4)	87 (9.3)	0.335	0.038
Pulmonary hypertension (%)	177 (4.3)	72 (7.7)	<0.001	0.142
Plus other heart surgery (%)	906 (22.1)	355 (37.9)	<0.001	0.349
Plus aorta surgery (%)	62 (1.5)	34 (3.6)	<0.001	0.134
Myocardial infarction (%)	900 (22.0)	343 (36.6)	<0.001	0.325
Septal perforation (%)	13 (0.3)	1 (0.1)	0.49	0.046
Ejection fraction (%)			<0.001	0.252
30−	290 (8.4)	121 (14.3)		
30–50	1088 (31.4)	312 (36.8)		
50+	2086 (60.2)	415 (48.9)		
Dialysis status (%)	17 (0.4)	7 (0.7)	0.189	0.044
Sepsis (%)	25 (0.6)	57 (6.1)	<0.001	0.308
Grafts (%)			<0.001	0.191
1 graft	730 (17.8)	214 (22.8)		
2 grafts	1584 (38.7)	317 (33.8)		
3 grafts	1238 (30.2)	264 (28.2)		
4 or more grafts	342 (8.4)	83 (8.9)		
Number of grafts unknown	199 (4.9)	59 (6.3)		
on-pump CABG (%)	3851 (94.1)	876 (93.5)	0.537	0.025

**Table 2:** Comparison of covariates in matched cohort.

Covariates	Non-FQ	FQ	*p*	SMD
*n*	821	821		
Male (%)	509 (62.0)	515 (62.7)	0.799	0.015
Age (mean (SD))	71.12 (10.24)	71.01 (10.80)	0.837	0.01
Admission type (%)			0.567	0.053
ELECTIVE	171 (20.8)	179 (21.8)		
EMERGENCY	628 (76.5)	626 (76.2)		
URGENT	22 (2.7)	16 (1.9)		
Smoking (%)			0.898	0.023
Active smoking	70 (8.5)	67 (8.2)		
No smoking	678 (82.6)	685 (83.4)		
Smoking history	73 (8.9)	69 (8.4)		
COPD (%)	136 (16.6)	130 (15.8)	0.738	0.02
Hyperlipemia (%)	435 (53.0)	421 (51.3)	0.521	0.034
Diabetes (%)	345 (42.0)	338 (41.2)	0.764	0.017
Hypertension (%)	489 (59.6)	489 (59.6)	1	<0.001
Infectious endocarditis (%)	4 (0.5)	7 (0.9)	0.547	0.045
Neurological dysfunction (%)	39 (4.8)	33 (4.0)	0.547	0.036
Extracardiac arteriopathy (%)	134 (16.3)	126 (15.3)	0.636	0.027
Angina (%)	225 (27.4)	229 (27.9)	0.869	0.011
Cardiac surgery history (%)	9 (1.1)	4 (0.5)	0.265	0.069
Coronary intervention history (%)	83 (10.1)	81 (9.9)	0.934	0.008
Pulmonary hypertension (%)	76 (9.3)	67 (8.2)	0.484	0.039
Plus other heart surgery (%)	311 (37.9)	312 (38.0)	1	0.003
Plus aorta surgery (%)	23 (2.8)	27 (3.3)	0.667	0.028
Myocardial infarction (%)	307 (37.4)	302 (36.8)	0.838	0.013
Septal perforation (%)	1 (0.1)	1 (0.1)	1	<0.001
Ejection fraction (%)			0.492	0.059
30−	132 (16.1)	115 (14.0)		
30–50	289 (35.2)	300 (36.5)		
50+	400 (48.7)	406 (49.5)		
Dialysis status (%)	4 (0.5)	6 (0.7)	0.753	0.031
Sepsis (%)	20 (2.4)	29 (3.5)	0.246	0.064
Grafts (%)			0.78	0.065
1 graft	198 (24.1)	186 (22.7)		
2 grafts	258 (31.4)	274 (33.4)		
3 grafts	224 (27.3)	232 (28.3)		
4 or more grafts	86 (10.5)	75 (9.1)		
Unknown number of grafts	55 (6.7)	54 (6.6)		
on-pump CABG (%)	755 (92.0)	765 (93.2)	0.397	0.046

**Table 3:** Cox regression in matched cohort (Covariates with a *p* value no more than 0.05 were included for multi-regression).

	Univariable Cox Regression			Multivariable Cox Regression		
Covariates	HR	*p*	CI	HR	*p*	CI
Floxacin	1.48	0.000	1.24–1.77	1.51	0.0000	1.26–1.81
Male	0.88	0.176	0.74–1.06			
Age	1.03	0.000	1.02–1.04	1.03	0.0000	1.02–1.04
Elective admission	1.2	0.125	0.95–1.51			
Emergency admission	1.12	0.636	0.69–1.82			
COPD	1.37	0.007	1.09–1.71	1.39	0.0047	1.11–1.75
Hypertension	0.73	0.000	0.61–0.87	0.84	0.0729	0.7–1.02
Neurological dysfunction	1.32	0.143	0.91–1.92			
Extracardiac arteriopathy	1.16	0.186	0.93–1.45			
Angina	0.66	0.000	0.53–0.81	0.85	0.1865	0.66–1.08
Cardiac surgery history	1.11	0.851	0.36–3.47	1.09	0.3982	0.89–1.34
Coronary intervention history	0.61	0.007	0.43–0.88	0.68	0.0406	0.47–0.98
Pulmonary hypertension	1.27	0.115	0.94–1.72			
Plus other heart surgery	1.28	0.007	1.07–1.53			
Myocardial infarction	1.25	0.013	1.05–1.5	1.12	0.3117	0.9–1.4
30% ≤ EF < 50%	1.02	0.902	0.8–1.29	0.97	0.7828	0.75–1.24
EF ≥ 50%	0.7	0.005	0.55–0.9	0.74	0.0228	0.58–0.96
Septal perforation	0.9	0.918	0.13–6.43			
Infectious endocarditis	0.78	0.725	0.19–3.13			
Plus aorta surgery	1	0.997	0.58–1.74			
Dialysis status	0.77	0.718	0.19–3.11			
Hyperlipemia	0.64	0.000	0.53–0.77	0.77	0.0068	0.63–0.93
Active smoking	1.33	0.157	0.89–1.99			
No smoking	1.06	0.834	0.63–1.79			
Diabetes	1	0.983	0.84–1.2			
Sepsis	1.89	0.001	1.31–2.73	1.68	0.0077	1.15–2.47
2 grafts	1.05	0.696	0.83–1.32			
3 grafts	0.84	0.186	0.66–1.09			
4 or more grafts	1.02	0.916	0.71–1.47			
Unknown number of grafts	1.09	0.638	0.76–1.56			

**Table 4:** Difference and their covariance of hospital stay, ICU stay, ventilation time, and vasopressors duration of the matched cohort.

		Hospital Stay	ICU Stay	Ventilation	Vasopressors Duration
FQ vs. non-FQ (paired *t* test, *p*)		<0.001	<0.001	<0.001	<0.001
Covariates (Covariance Test, *p*)	Gender (Male)	0.792	0.630	0.409	0.792
	COPD	0.755	0.500	0.106	0.755
	Neurological dysfunction	0.130	0.016	<0.001	0.130
	Extracardiac arteriopathy	0.366	0.263	0.671	0.366
	Angina	0.901	0.472	0.701	0.901
	Cardiac surgery history	0.394	0.339	0.044	0.394
	Coronary intervention history	0.718	0.796	0.468	0.718
	Pulmonary hypertension	0.810	0.152	0.305	0.810
	Plus other heart surgery	0.135	0.050	0.046	0.135
	Myocardial infarction	0.727	0.667	0.881	0.727
	Ejection fraction	0.612	0.958	0.590	0.612
	Admission type	0.560	0.953	0.823	0.560
	Septal perforation	0.854	0.634	0.580	0.854
	Infectious endocarditis	0.796	0.009	0.712	0.796
	Plus aorta surgery	0.033	0.000	<0.001	0.033
	Dialysis status	0.726	0.971	0.521	0.726
	Age	0.490	0.818	0.423	0.490
	Hyperlipemia	0.005	0.001	0.135	0.005
	Smoking	0.193	0.089	0.182	0.193
	Diabetes	0.623	0.416	0.626	0.623
	Sepsis	0.257	0.208	0.416	0.257

**Table 5:** Difference and their covariance of in hospital death, post-operative stroke, and IABP application of the matched cohort (logistic regression).

	In Hospital Death	Post-Operative Stroke	IABP
FQ	0.0055	0.0124	0.0011
Male	0.1747	0.0066	0.0683
Age	0.0072	0.0591	0.0022
Elective admission	0.9407	0.9909	0.0065
Emergency admission	0.3858	0.7185	0.0574
No smoking	0.9844	0.9369	0.9913
Smoking history	0.9855	0.8746	0.4540
Dialysis status	0.9954	0.3650	0.2139
Hyperlipemia	0.4447	0.0288	0.3194
Diabetes	0.8175	0.3973	0.0381
COPD	0.8013	0.2617	0.4999
Neurological dysfunction	0.1964	0.0000	0.1151
Extracardiac arteriopathy	0.0000	0.3843	0.0077
Angina	0.7161	0.8917	0.0797
Myocardium infarction	0.0066	0.2593	0.0000
Cardiac surgery history	0.9955	0.9900	0.2738
Coronary intervention history	0.2402	0.6902	0.9584
Pulmonary hypertension	0.3829	0.4057	0.0197
Septal perforation	0.9984	0.9963	0.5800
Plus other heart surgery	0.4736	0.1292	0.8322
EF 30–50	0.2449	0.7837	0.0000
EF 50+	0.0740	0.9964	0.0000
Sepsis	0.0000	0.0259	0.1294
Hypertension	0.0070	0.2375	0.0085
on-pump CABG	0.0426	0.3812	0.7707
2 grafts	0.1016	0.4998	0.6797
3 grafts	0.2520	0.1353	0.3002
4 or more grafts	0.7899	0.3423	0.9350
Unknown number of grafts	0.7755	0.3209	0.2536

## Data Availability

All original data are available at reasonable request via email: huzhipengphd@whu.edu.cn.
